# Contiguity and overshadowing interactions in the rapid-streaming procedure

**DOI:** 10.3758/s13420-023-00582-4

**Published:** 2023-04-17

**Authors:** José A. Alcalá, Ralph R. Miller, Richard D. Kirkden, Gonzalo P. Urcelay

**Affiliations:** 1 School of Psychology, University of Nottingham, University Park, Nottingham, NG7 2RD UK; 2 Departamento de Psicología Experimental, Procesos Cognitivos y Logopedia, Universidad Complutense de Madrid, Madrid, Spain; 3 Department of Psychology, State University of New York at Binghamton, Binghamton, NY USA; 4 Department of Neuroscience, Psychology and Behaviour, University of Leicester, Leicester, UK

**Keywords:** Temporal contiguity, Overshadowing, Cue competition, Rapid trial streaming

## Abstract

When multiple cues are associated with the same outcome, organisms tend to select between the cues, with one revealing greater behavioral control at the expense of the others (i.e., cue competition). However, non-human and human studies have not always observed this competition, creating a puzzling scenario in which the interaction between cues can result in competition, no interaction, or facilitation as a function of several learning parameters. In five experiments, we assessed whether temporal contiguity and overshadowing effects are reliably observed in the streamed-trial procedure, and whether there was an interaction between them. We anticipated that weakening temporal contiguity (ranging from 500 to 1,000 ms) should attenuate competition. Using within-subject designs, participants experienced independent series of rapid streams in which they had to learn the relationship between visual cues (presented either alone or with another cue) and an outcome, with the cue-outcome pairings being presented with either a delay or trace relationship. Across experiments, we observed overshadowing (Experiments 1, 2, 4, and 5) and temporal contiguity effects (Experiments 2, 3, and 4). Despite the frequent occurrence of both effects, we did not find that trace conditioning abolished competition between cues. Overall, these results suggest that the extent to which contiguity determines cue interactions depends on multiple variables, some of which we address in the *General discussion*.

**Supplementary Information:**

The online version contains supplementary material available at 10.3758/s13420-023-00582-4.

## Introduction

When events co-occur, contiguity – that is temporal and spatial proximity – largely determines whether organisms encode the relationship between them. The closer in time and space they are experienced, the stronger the relationship between them that is learned. After repeated presentations, the antecedent event (hereafter, the cue [C]) becomes a reliable predictor of the second event (hereafter, the outcome [O]), producing changes in behavioral control by the cue, ordinarily in anticipation of the outcome. However, when the appearance of the outcome is delayed in time, non-human and human animals are remarkably sensitive to these variations, showing decreased behavioral control by the cue as the interval between the cue and the outcome increases (Dickinson et al., 1992; Dignath et al., 2018; Greville & Buehner, 2010; Reynolds, 1945; Shanks & Dickinson, 1991; Stephens & Kalish, 2018). Although the effect of temporal contiguity has been a matter of great interest since the very origin of learning research (see Boakes & Costa, 2014, for a review), several findings in the 1960s produced a shift in interest from contiguity to contingency, driven (among other findings) by cue-interaction phenomena such as Kamin’s blocking effect (see Wasserman & Miller, 1997, for a review).

Cue-interaction phenomena refer to situations in which the behavioral control acquired by a target cue X is modified by the presence of additional cues during training. Probably, the most influential phenomenon of cue interactions is blocking (Dickinson et al., 1984; Kamin, 1969). In blocking, the target cue X is trained in compound with the blocking cue A, which has received additional training, either before the compound training (i.e., forward blocking), after the compound training in a retrospective re-evaluation design (i.e., backward blocking), or with trials of A-O and AX-O interspersed (i.e., single-phase blocking). In each of these situations, the target cue X often shows less behavioral control compared to a control condition in which X is trained in the presence of a neutral stimulus (i.e., B). Although blocking is usually considered a canonical example of cue competition (A “competes” with X for behavioral control), it is not a *pure* cue competition phenomenon, primarily because the competing cue A is trained separately from the compound. This potentially introduces associative interference in addition to associative competition as evidenced by forward and backward blocking being sensitive to contextual changes from training to test, akin to the renewal effect observed in associative cue interference (Miguez & Miller, 2022). A *purer* example of cue competition is overshadowing (Pavlov, 1927). A typical overshadowing design involves training a compound of cues (AX) and then comparing the response to the target cue (X) of a control condition in which a control cue (i.e., Y) was trained alone. Results typically reveal decreased behavioral control by X compared to Y. Unlike the blocking design, the cues that are compared have identical training histories.

A critical implication of cue-competition phenomena is that contiguity is not sufficient for behavioral control (i.e., learning) to occur. Although in these designs contiguity between target cue and outcome is identical to contiguity between the control cue and the outcome, reduced behavioral control by the target cue is observed. This has had a profound impact on the way theorists conceptualize learning. Therefore, cue-competition phenomena have become a benchmark for the majority of models of learning (e.g., Dickinson & Burke, 1996; Pearce, 1987; Pearce & Hall, 1980; Rescorla & Wagner, 1972; Stout & Miller, 2007; Van Hamme & Wasserman, 1994; Wagner, 1981).

The particular case of overshadowing has received a lot of attention and is consistently observed across species (including invertebrates) and learning protocols (Buckley et al., 2021; Buckley et al., 2019; Ghinescu et al., 2016; Kattner & Green, 2015; Pearce & Hall, 1978; Prados, 2011; Stahlman et al., 2018). Nevertheless, despite the generality of overshadowing phenomena, it is also clear that the presence or absence of overshadowing depends on specific parameters, variables, and learning protocols (see Wheeler & Miller, 2008). Critically, one of these variables is cue-outcome temporal contiguity (Herrera et al., 2022; see Urcelay, 2017).

In the case of overshadowing, when temporal contiguity between the cues and the outcome is manipulated, there is an asymmetrical effect on the behavioral control of the target cue trained in compound relative to when it is trained alone. That is, when conditioning occurs with strong contiguity, competition between cues has been reported (i.e., the overshadowing cue weakens behavioral control by the target cue, overshadowing). However, reduced temporal contiguity leads to the absence of overshadowing and sometimes even the opposite, that is, facilitation between cues is occasionally observed (i.e., the overshadowing cue potentiates behavioral control by the target cue, that is, potentiation). This pattern of results has been observed in animals using fear conditioning (Urcelay & Miller, 2009), taste aversion (Batsell et al., 2012), and instrumental learning (Schachtman et al.,^1987^). In recent years, some studies exploring this interaction in humans, using predictive (Cunha et al.,^2015^; Herrera et al., 2022) and action-outcome (i.e., instrumental) learning tasks (Alcalá et al., 2023), have also documented this cross-over interaction. For instance, Herrera et al. (2022) explored overshadowing as a function of temporal contiguity in an avoidance-learning task. With strong contiguity (i.e., delay conditioning), they reported a robust overshadowing effect across experiments. However, inserting a temporal gap between the offset of the cues and the onset of the aversive outcome (i.e., trace conditioning) decreased overshadowing, resulting in no competition between cues (also see Alcalá et al., under review). The robustness of the null effect was corroborated by Bayesian analyses, supporting the absence of interaction under those conditions and suggesting that the cue-outcome trace interval attenuated competition, but was not sufficient to produce facilitation. Alcalá et al. (2023), using an action-outcome learning task, observed that an intervening signal (i.e., overshadowing cue) competed with the instrumental action when there was strong temporal contiguity between action and outcome (2-s trace interval), but the very same intervening signal facilitated the instrumental action with weak temporal contiguity (6-s trace interval).

The pattern of results from the interaction between contiguity and overshadowing procedures is consistent with the view that there is a continuum in cue-interaction phenomena, with competition and facilitation being two extremes of this continuum (see Urcelay, 2017). One extreme of the continuum is overshadowing (i.e., competition) and the other extreme is potentiation (i.e., facilitation), with no interaction in the middle.

The description of competition as one extreme of a continuum is particularly fitting in light of recent results that suggest that cue competition is not as universal as previously thought. Although overshadowing and blocking are well-established phenomena in the literature, there are numerous examples in which no cue competition was observed in different learning preparations and species (e.g., Beesley & Shanks, 2012; Bott et al.,^2007^; Maes et al., 2016; Murphy & Dunsmoor, 2017; Schmidt & De Houwer, 2019; Waldmann, 2001). The absence of competition is not entirely surprising because cue-interaction phenomena are sensitive to several experimental parameters (see Wheeler & Miller, 2008). However, the failures to observe cue competition are informative concerning the boundary conditions of the behavioral determinants that yield competition or facilitation (Urcelay, 2017), as well as the generality of such learning phenomena (Maes et al., 2018; Seraganian, 2022; Soto, 2018). Given that cue-competition phenomena have received a vast amount of attention in the learning literature, it is not surprising that previous research has revealed several variables and parameters that modulate whether competition or facilitation between cues is observed. In addition to temporal contiguity, there are other variables affecting competition such as contingency (Urcelay & Miller, 2006; Urushihara & Miller, 2007), relative stimulus duration (Sissons et al., 2009), configural versus elemental encoding of the cues (Williams & Braker, 1999; Williams et al., 1994; also see Melchers et al., 2008), number of trials and inter-stimulus interval (Bellingham & Gillette, 1981; Stout et al., 2003a; Stout et al., 2003b), the framing of the test question (Pineño et al., 2005), and the presence of a secondary task (De Houwer & Beckers, 2003), to cite just a few factors. For instance, Vadillo and Matute (2010, 2011) found that time pressure on participants’ responding, which presumably increases task demands, is a critical factor in determining competition or facilitation in a blocking design. When participants had appreciable time to respond, a clear blocking effect was observed. However, when participants were trained under time pressure, the blocked cue yielded *more* behavioral control than the control cue – that is, augmentation. Note that the continuum from blocking-to-augmentation is equivalent to the continuum from overshadowing-to-potentiation, with competition at one end of the continuum and facilitation at the other. The authors’ interpretation was that the time pressure depleted participants’ cognitive resources, thereby promoting a reduction in competition between cues.

In the present series, we used a task in which the training trials were presented very rapidly, and consequently might favor an interaction between overshadowing and contiguity. Moreover, given the considerable number of variables that are known to affect cue-interaction phenomena, we used a paradigm in which multiple independent variables could be manipulated within subjects to identify the boundaries of any potential interaction. Hence, we adopted the streamed-trial procedure developed by Crump et al. (2007).

In the streamed-trial procedure, trials are typically presented for a very brief time (e.g., 100 ms) separated by a short inter-trial interval (ITI; e.g., 100 ms). During each stream of trials, the relationship between the cues and outcomes can be manipulated in accord with a 2 × 2 contingency matrix in which the cue(s) C (present or absent) covary with the outcome O (present or absent), resulting in four different trial types: type ‘a’ C|O, type ‘b’ C|~O, type ‘c’ ~C|O, type ‘d’ ~C|~O. After each stream, a question is presented that assesses the strength of relatedness between the target cue and the outcome. This arrangement speeds experiments assessing the detection of covariation between stimuli, thereby allowing each participant to be exposed to multiple streams with the critical factors manipulated across streams. This protocol has been used previously to assess contingency judgments (e.g., Allan, Hannah, Crump, & Siegel, 2008; Castiello et al., 2022; Crump et al., 2007; Jozefowiez, 2021; Maia et al., 2018). More directly related to our concerns, this task has also proved effective in the detection of forward, backward, and single-phase blocking (Hannah et al., 2009; Mutter & Arnold, 2020); thus, it is a sensitive procedure to explore cue-interaction phenomena using statistically powerful within-subject designs.

In summary, the aims of the current experimental series were twofold. First, we wanted to assess whether people are sensitive to overshadowing and contiguity manipulations in a rapid stream of trials. To the best of our knowledge, there are no reports in the literature of overshadowing or contiguity effects using this procedure. Second, we wanted to evaluate the potential interaction between the two phenomena. We expected to observe overshadowing with strong temporal contiguity, but no interaction or perhaps potentiation with weak temporal contiguity.

Table 1 summarizes the experimental designs and parameter manipulations used in each of the experiments reported here. Experiment 1 was designed to determine whether overshadowing can be obtained using a rapid stream of trials and whether the magnitude of the overshadowing effect depends on the number of training trials. Having established overshadowing, Experiment 2 sought a contiguity effect, comparing delay conditioning (i.e., cue offset coincided with outcome onset) with trace conditioning (a 500-ms interval was introduced between cue offset and outcome onset) as well as varying the number of trials. The factorial arrangement of contiguity and overshadowing here was intended to facilitate the detection of any potential interaction between these factors. Experiment 3 assessed this interaction using a somewhat longer delay interval (800 ms). Experiments 4 and 5 further assessed this interaction but using a smaller number of trials. Anticipating the results, both contiguity and overshadowing effects were observed in most experiments, although they proved sensitive to parametric variations. Across experiments, the expected cross-over interaction between cue interaction and contiguity was consistently absent.

**Table 1:** Experimental designs and parameters

Exp.	n	Contiguity	Contingency	ITI	Training trials	Filler trials	
1	29	0 ms	High	600 ms	16 vs. 32	8	
2	30	0 vs. 500 ms	High	600 ms	16 vs. 32	8	
3	25	0 vs. 800 ms	High	1,200 ms	32	8	
4	26	0 vs. 1,000 ms	High	1,200 ms	12	12	
5	29	0 vs. 500 ms	High vs. Low	600 ms	12	12	

Summary of the experiments and critical manipulations conducted in each experiment (Exp.). ITI is intertrial interval and n is the number of participants. In each experiment, one target cue was always presented in compound with a potentially interactive nontarget cue and one control target cue was presented alone

## Experiment 1

We assessed whether overshadowing would be observed using a rapid stream of brief events. Using the streamed-trial procedure, participants were presented with a rapid stream of cue-outcome pairings and then we assessed participants’ judgments of the relatedness between them. In compound streams, a simultaneous compound of two cues (target cue and overshadowing cue) was presented and paired with the outcome, whereas in the control streams, a single cue was paired with the outcome. Because all participants experienced all types of streams, each participant served as their own control to evaluate overshadowing. We expected to observe lower judgments of relatedness between the target cue (i.e., overshadowed) and the outcome, compared to the control cue that was trained alone.

We additionally manipulated the number of training trials. Previous research has suggested that the number of trials modulates the magnitude of the overshadowing effect. For instance, rodent studies have revealed that the overshadowing effect is attenuated with an increase in the number of trials (Bellingham & Gillette, 1981; Stout et al., 2003a). Studies using human participants have reported that over-selectivity (a phenomenon somewhat similar to overshadowing) is reduced by increasing the number of training trials (Reed & Quigley, 2019). However, in a recent study, we did not find a clear effect of the number of trials across experiments (Herrera et al., 2022). Hence, we examined two different numbers of training trials. In half of the streams, there were 16 training trials, whereas in the other half, there were 32 training trials. A priori, we expected to observe a stronger overshadowing effect in the condition with 16 training trials.

### Method

#### Participants

Forty participants (six females and 34 males) were recruited from Amazon Mechanical Turk (MTurk). Their mean age was 34.9 years, *SD* = 6.30 (min = 24, max = 48). The original study of Crump et al. (2007) in which *n* = 37 was used as a reference to estimate the necessary sample size. Participants received US$3.50 as compensation. All participants declared they were not prone to visually induced seizures. Participants were required to be based in the USA, Canada, UK, New Zealand, or Australia and be 18–50 years old. In addition, they needed to have completed more than 500 prior approved MTurk Human Intelligence Tasks (HITs) with a mean approval ≥ 98%. Participants could take part in this series only once. These criteria were applied in this and all subsequent experiments. After applying the exclusion criteria detailed below, 29 participants (five females and 24 males) were included in the analyses (mean age = 35.6 years, *SD* = 6.54). Each experiment was approved by the Ethics Committee at either the University of Leicester or the University of Nottingham.

#### Design

A 2 (Cue: Control vs. Target) × 2 (Training Trials: 16 vs. 32) factorial design was used. The different conditions were presented in an order randomized for each participant. In some streams, the control cue was trained alone, while in other streams the target cue was presented in the presence of the overshadowing cue. After each stream, we evaluated the degree of relatedness between the cue (the control, the target, or the overshadowing cue) and the outcome. In streams in which a compound of cues was presented, the target cue was tested for some streams and the overshadowing cue was tested for others (only half as many times as the target cue) to prevent participants from learning that only one of the two cues of the compound would be tested. However, considering the goal of the experiments and for ease of exposition, we present only the results for the target cue. Because all streams occurred with a delay conditioning procedure, strong overshadowing was expected.

#### Materials and apparatus

The task was programmed and hosted using the Gorilla Experiment Builder (Anwyl-Irvine et al.,^2020^). The screen background was black (RGB: 0, 0, 0) and all stimuli were presented against the same background. We used different geometrical shapes in grey (RGB: 127, 127, 127) as target and control cues. These shapes measured approximately 95 × 95 pixels. The same geometrical shapes, but larger and in a black and white pattern, were used as overshadowing cues. Overshadowing cues measured approximately 130 × 130 pixels. The exact size of the stimuli depended on the geometrical shape used. Stimuli were displayed at their original size; however, the stimuli occupied different proportions of the screen depending on the size of the participant´s screen. Based on the average Gorilla participant´s screen size reported in a large sample of studies conducted online (Anwyl-Irvine et al.,^2021^), the control/target and overshadowing cues occupied around 11.4% (± 1 SD: 10.2–13.0%) and 15.6% (± 1 SD: 14.0–17.7%) of the participant´s screen height, respectively.

Outcomes were common objects represented in white. These objects occupied 11.4% (± 1 SD: 10.2–13.0%) of the participant´s screen height and always appeared below the centered fixation cross. The cue-outcome dyads were predefined before the experiment so that all participants received the same combination of cue-outcome dyads. The only constraint in forming these dyads was that cues did not have the same shape within a given stream (e.g., a white square as the target cue and a stripped square as an overshadowing cue would not be paired). During the experiment, each cue-outcome dyad was only used once, and hence in each stream, all stimuli used were new to participants.

#### Procedure

After reading and signing the consent form, participants were presented with the instructions:

- *In this experiment, you will be watching numerous sequences. In each sequence, you will be presented with a rapid series of different SHAPES (e.g., a square and/or a triangle) and different PICTURES (e.g., a ball or a book). The shapes and pictures may or may not occur together. This way, during each sequence, there are three types of events that can occur. (1) The shape and the picture occur together. (2) The shape appears, but the picture does not appear. (3) The shape does not appear, but the picture appears.*
- *Each sequence that you see will contain many of each of these three types of events. After each sequence, a question screen will appear and you will be asked to rate the degree of relatedness between the shape and the picture. In other words, you are evaluating the extent to which the shape anticipates or predicts the appearance of the picture. You will have to evaluate this relationship on a scale from -10 to 10.*
- ***POSITIVE RATINGS*** *should be given when presentation of the shape was soon followed by presentation of the picture. In other words, most of the time the shape appeared together with the picture. That is, the shape predicted the impending appearance of the picture.*
- ***NEGATIVE RATINGS*** *should be given when presentation of the shape was NOT soon followed by presentation of the picture. In other words, most of the time the shape appeared without the picture; thus, the picture and shape were NOT presented together. That is, the shape predicted the absence of the impending appearance of the picture.*
- *To complete this HIT and be compensated, you must give your undivided attention to the task for a total of approximately 30 minutes (excluding breaks). During the experiment, a series of sequences of images will be presented on your screen, with each sequence running for less than a minute. Please keep your eyes on the cross that will appear in the center of the screen during each sequence. The shapes, if any, will always appear above the cross, whereas the picture, if any, will always appear below the cross. Immediately after each sequence, a simple, one-click question will be asked concerning the sequence that you just saw. The strength of your rating should reflect your perception of the relationship during each sequence. Keep in mind that the relationship between the shape and picture may change across sequences.*
- *You must answer each question to the best of your ability within 10 seconds of its being presented. Failure to respond to each of these questions within 10 seconds will result in termination of the HIT without compensation. After you enter your answer, there will be the opportunity for you to take a break of up to 10 minutes before you must start the next sequence. If you do not initiate the next sequence within 10 minutes, your participation will be terminated without compensation. If these conditions are unacceptable to you, please withdraw from this HIT.*

**Fig. 1:**
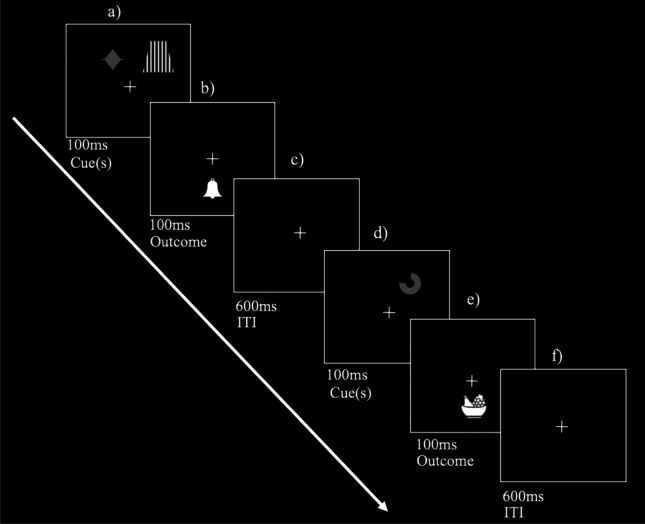
Schematic representation of a trial in a compound stream with delay conditioning. Panel (**a**) illustrates the cues for a training trial in which the target cue (smooth diamond) and the overshadowing cue (striped trapeze) appear above the fixation cross; note that in a control stream only the control cue (a smooth figure) would appear. Panel (**b**) illustrates the outcome paired with the compound. Panels (**c**) and (**f**) represent the inter-trial interval (ITI) in which only the fixation cross was present on the screen. Panel (**d**) illustrates the cue for a single filler trial; note that the filler cue can appear alone or in the presence of a filler overshadowing cue. Panel (**e**) illustrates the outcome paired with the filler cues. The training trials (Panels **a** and **b**) and filler trials (Panels **d** and **e**) were randomly intermixed within each stream

After reading the instructions, the experiment started. As depicted in Fig. 1, each stream was displayed over a solid black background. There was a white-cross fixation point at the center of the screen. The cross was present throughout each stream.

Cues always appeared above the fixation cross and switched between left and right positions without any particular restriction in the order that they appeared (e.g., left, right, left, left, left, right…), but with the constraint that they would appear a similar number of times on the left and right positions (e.g., six times on the left and six times on the right). Cue(s) were presented for 100 ms on the screen without the outcome present (Fig. 1, Panel a). Immediately after the offset of the cue(s), the outcome was always displayed below the fixation cross (Fig. 1, Panel b) for 100 ms (i.e., Delay Condition). There was an ITI during which only the fixation cross appeared, which lasted 600 ms (Fig. 1, Panel c).

There were two types of streams. In the single streams, the control cue was paired with the outcome. In the compound streams, the target cue was presented simultaneously with the overshadowing cue (Fig. 1, Panel a), and both were paired with the outcome (Fig. 1, Panel b). The control and target cues were presented for a pre-determined number of times (i.e., training trials), 16 times in the 16-trial condition and 32 times in the 32-trial condition. The cue-outcome contingency was imperfect: on 75% of the training trials the target or control cues were followed by the outcome (i.e., type ‘a’ trials), and on 25% of the trials the cue appeared but was not followed by the outcome (i.e., type ‘b’). Thus, in the 32-trial condition, there were 24 type ‘a’ trials and eight type ‘b’ trials, whereas, in the 16-trial condition, there were 12 ‘a’ trials and four ‘b’ trials.

In addition to the training trials, each stream contained eight filler trials. For the filler trials, cues and outcomes were of the same generic nature (e.g., geometrical shapes and objects) but of different kinds than the cues and outcomes used on the training trials of that stream. In each stream, there was one filler cue (Fig. 1, Panel d) presented by itself (i.e., no other cue present) and paired with a non-target outcome (Fig. 1, Panel e) half of the time. The same cue appeared in the presence of a filler overshadowing cue (e.g., a stripped circle) paired again with the same non-target outcome in the other half of trials. In all the streams, there were eight filler trials, consisting of four single filler trials and four compound filler trials. Note that training and the filler trials were randomly intermixed during each stream without any restrictions. The same contingency ratio applied to the filler cues. Of the eight filler trials, six were ‘a’ trials (three single filler trials and three compound filler trials) and two were ‘b’ trials (one single filler trial and one compound filler trial). The number of filler trials was independent of the number of training trials. Filler cues were never tested. Thus, in the streams with 16 training trials, there were 24 trials (16 training trials + 8 filler trials), and in the streams with 32 training trials, there were 40 trials (32 training trials + 8 filler trials).

After each stream, a single test question was presented with an image of the cue and outcome being tested, asking participants to evaluate the relatedness of the cue and the outcome with the following instructions: “*Please, indicate the degree of relatedness between [picture of the cue] and the [picture of the outcome] in the series that you just saw. Use the rating scale below to enter the degree of relatedness.*” There was a rating scale, ranging from -10 (strongly negatively related) to 10 (strongly positively related) presented beneath the cue and outcome. The pointer was positioned at 0 on the scale, and participants could move the pointer with the mouse in either direction. A video example of a stream with Delay conditioning is available in the Open Science Framework (OSF) link.

After participants answered the test question, a screen was presented that instructed participants that they could take a break, of no more than 10 min. Participants needed to press the space bar to start the next stream. If participants did not start the next stream within the allowed 10 min, the participant was terminated with the data being discarded.

Overall, participants experienced 50 different streams: 20 streams training and testing the control cue, 20 streams training a compound and testing the target cue, and 10 streams training a compound and testing the overshadowing cue. The 50 streams were divided into five blocks of ten streams (see Online Supplementary Material (OSM) for the exact distribution of streams in a block). The duration of the streams was 32 s and 19 s for the 32-trial and 16-trial conditions, respectively.

#### Data exclusion

In all experiments, three different attentional checks were implemented to ensure data quality: time limit during test questions, attentional check during a bogus test question (in Experiment 5 we introduced a contingency-awareness check rather than this attentional check), and a question concerning subjective commitment by participants.

- Participants had a maximum of 20 s to respond to test questions. If participants did not respond within this limit, they were not allowed to continue with the experiment. Consequently, all participants who finished the task always made a response within the limit of 20 s. The instructions stated that participants had a maximum of 10 s; however, the program allowed twice that time. This time constraint was intended to maximize the attention during the stream of trials, and minimize the likelihood of streams running on their screens without them paying attention.
- After running the 50 streams used in the experimental design, a final bogus stream was run. This stream was similar to the previous 50 streams, with the exception that we introduced an attentional check during the test question. Instead of the two images representing the typical layout of the cue and outcome during the test, this attentional screen showed a single picture with four white ovals and the sentence “*Please indicate the numbers of ovals in the image below using the rating scale.”* For all experiments, we considered that participants had passed the attentional check if they selected 3, 4, or 5 in the slider. We allowed one value below or above the correct response to permit some inaccuracies whilst moving the slider. Participants who selected a different response were eliminated from all analyses, and the data of these participants were not replaced.
- After the attentional check, we directly asked participants about their subjective commitment during the task with the following question:

- *Well done! The experiment is over. Just one last question. Did you give your full attention to the experimental task (as opposed to sometimes doing other things like using your smartphone) while stimuli were being presented? Please, answer honestly; this question has no impact on your payment. There are two options below “Yes” and “No.”*

Participants who selected *“No”* were removed from the analyses and the data of these participants were not replaced.

After applying these attentional checks, 11 participants failed to respond correctly to the attentional check, and consequently only 29 participants were considered for data analyses. Furthermore, sensitivity analyses using the software G*power revealed that with a final sample of 29 participants, the smallest effect size that could be detected for the main effect of Cue with a power of .80 and an alpha criterion of .05 was η^*2*^_*p*_ = .06. Note that this effect is considerably smaller than the observed effect size (η^*2*^_*p*_ = .51, see *Results*).

#### Data analyses

A 2 (Cue: Control vs. Target) × 2 (Training Trials: 16 vs. 32) repeated-measures ANOVA was conducted. The mean ratings across streams in each experimental condition for each participant were analyzed. An Overshadowing Index was calculated by subtracting the mean rating of the target cue (i.e., trained as part of a compound) from that of the control cue to assess overshadowing between conditions. Positive values represented overshadowing, a value of 0 indicated no overshadowing, and negative values reflected potentiation. A rejection criterion was set at .05 for all statistical tests. Partial eta-squared measures served as effect sizes and their 95% confidence intervals (CIs) were reported using the software available in Nelson (2016). Additionally, Cohen´s *d* and their 95% CIs were reported using the JASP 0.15 software (JASP, 2021). Although we collected data for streams in which the overshadowing cue was tested, those data were not analyzed (however, these ratings are available in the archived data set).

### Results

**Fig. 2:**
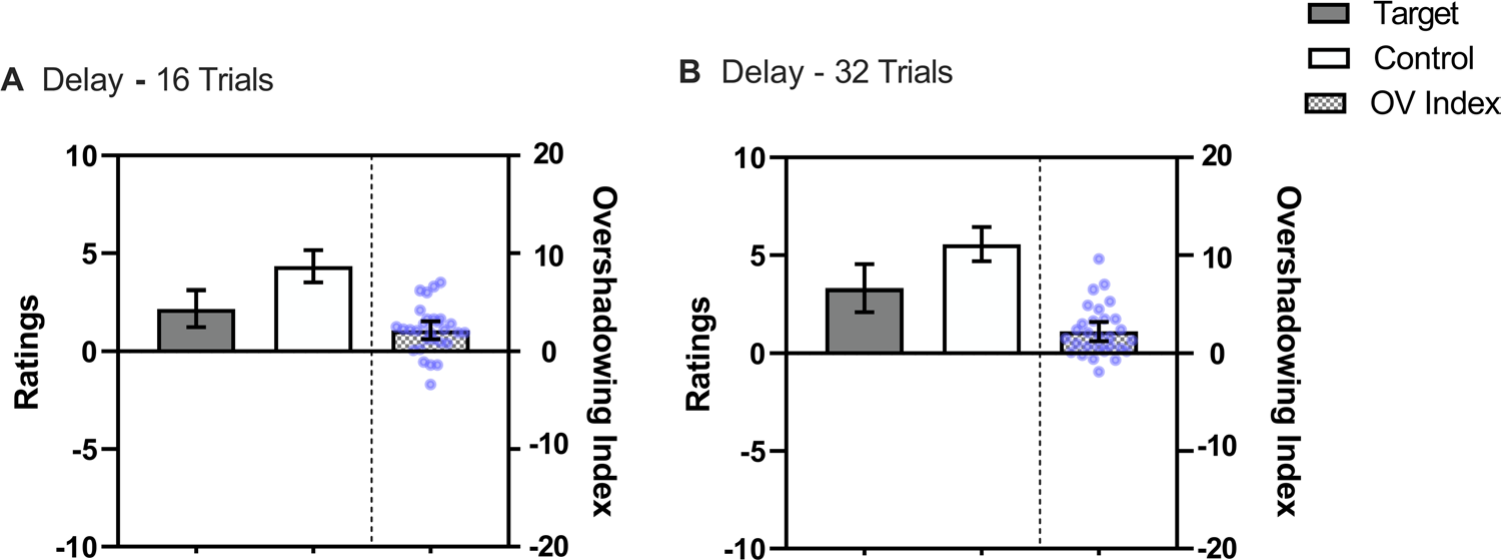
Mean ratings across streams in Experiment 1 for the Target cue which was trained as part of a compound and the Control cue which was trained alone. The checkered bars represent the Overshadowing Index (Control Cue – Target Cue); values above zero indicate Overshadowing and a value of 0 represents no competition. The blue points represent each participant’s Overshadowing Index. Panel **A **displays the ratings for 16 trials and Panel **B** for 32 trials. Error bars represent 95% confidence intervals

Figure 2 depicts mean ratings for control and target cues (left side of panels) as a function of the number of training trials, and also the Overshadowing Index (right side of panels). Not surprisingly, 16 trials resulted in lower ratings than 32 trials. More central to our concerns, with both 16 and 32 target training trials, the Overshadowing Index was above 0, indicative of overshadowing. A repeated-measures 2 (Cue: Control vs. Target) × 2 (Trials: 16 vs. 32) ANOVA confirmed these impressions, revealing a main effect of Trials, *F*(1, 28) = 20.36, *p* < .001, η^*2*^_*p*_ = .42, 95% CI [.14, .60], a main effect of Cue, *F*(1, 28) = 29.62, *p* < .001, η^*2*^_*p*_ = .51, 95% CI [.23, .67], but not an interaction, *F*(1, 28) = 0.03, *p* = .865.

Increasing the number of training trials increased ratings of relatedness, suggesting that the frequency of trials is critical to detect the relationship between cues and outcomes (see Murphy et al., 2022). But the absence of an interaction suggests that the number of trials did not alter the magnitude of the overshadowing effect. Thus, a clear example of overshadowing was observed using our adaptation of the streamed-trial procedure (Crump et al., 2007), but it was not modulated by the number of training trials.

## Experiment 2

The second experiment was intended to directly address the interaction between contiguity and overshadowing. Half of the streams were trained similarly to that of Experiment 1 with delay conditioning, but the remaining half were presented with a trace interval between the cue(s) and outcome, specifically, with a 500-ms interval interposed between the offset of the cue and the onset of the outcome.

In a preliminary study using a 500-ms trace, we observed a reliable effect of contiguity (i.e., lower ratings in the trace condition compared to the delay condition). Based on this preliminary experiment, in Experiment 2 we expected to observe a weakening of behavioral control as temporal contiguity decreased in the control cue (trained in a single-cue stream). Furthermore, we anticipated that trace conditioning would impact the target cue differently when trained in a compound, and therefore we expected that competition would be at least attenuated (i.e., less overshadowing) in the trace condition streams, relative to the delay condition streams (Herrera et al., 2022; Urcelay & Miller, 2009).

### Method

#### Participants

Forty participants (13 females and 27 males) recruited from MTurk took part. Their mean age was 36.7 years, SD = 7.4 (min = 18, max = 49). The same participation requirements as in Experiment 1 were applied. Participants received US$4.00 as compensation. In the attentional check, ten participants failed to respond correctly, consequently, data from 30 participants were used in the subsequent analyses (ten females and 20 males; mean age = 36.86 years; *SD* = 7.25)

#### Design and procedure

A 2 (Trials: 16 vs. 32) × 2 (Contiguity: Delay vs. Trace) × 2 (Cue: Control vs. Target) within-subjects design was used. The same stimuli and procedures as in Experiment 1 were used, except for the inclusion of trace as a factor and the overall number of streams presented. Delay conditions were identical to Experiment 1 (0 ms between the offset of the cue/s and the onset of the outcome). In trace conditions, there was a 500-ms interval between the offset of the cue/s and onset of the outcome. During the trace interval, the fixation cross remained present on the screen. The contiguity factor was counterbalanced across streams. For example, stream 1 was presented with delay conditioning for half of the participants and with trace conditioning for the other half, while stream 2 was presented with trace conditioning for half of the participants and with delay for the other half. Hence, all streams were rated within delay and trace conditioning conditions. Filler trials, in all streams (i.e., delay and trace conditions), were half delay and half trace. A video example of a stream with trace conditioning is available in the OSF link.

There were 40 streams in this experiment. In 16 streams the control cue was tested, in another 16 streams the target cue was tested, and in eight streams the overshadowing cue was tested. The 40 streams were divided into four blocks of ten training streams (see OSM for more details). In the 32 training trial conditions, the duration of each stream was 32 s in the delay conditions and 52 s in the trace conditions, and in the 16 training trial conditions, 19 s in the delay conditions and 31 s for the trace conditions.

### Results

**Fig. 3:**
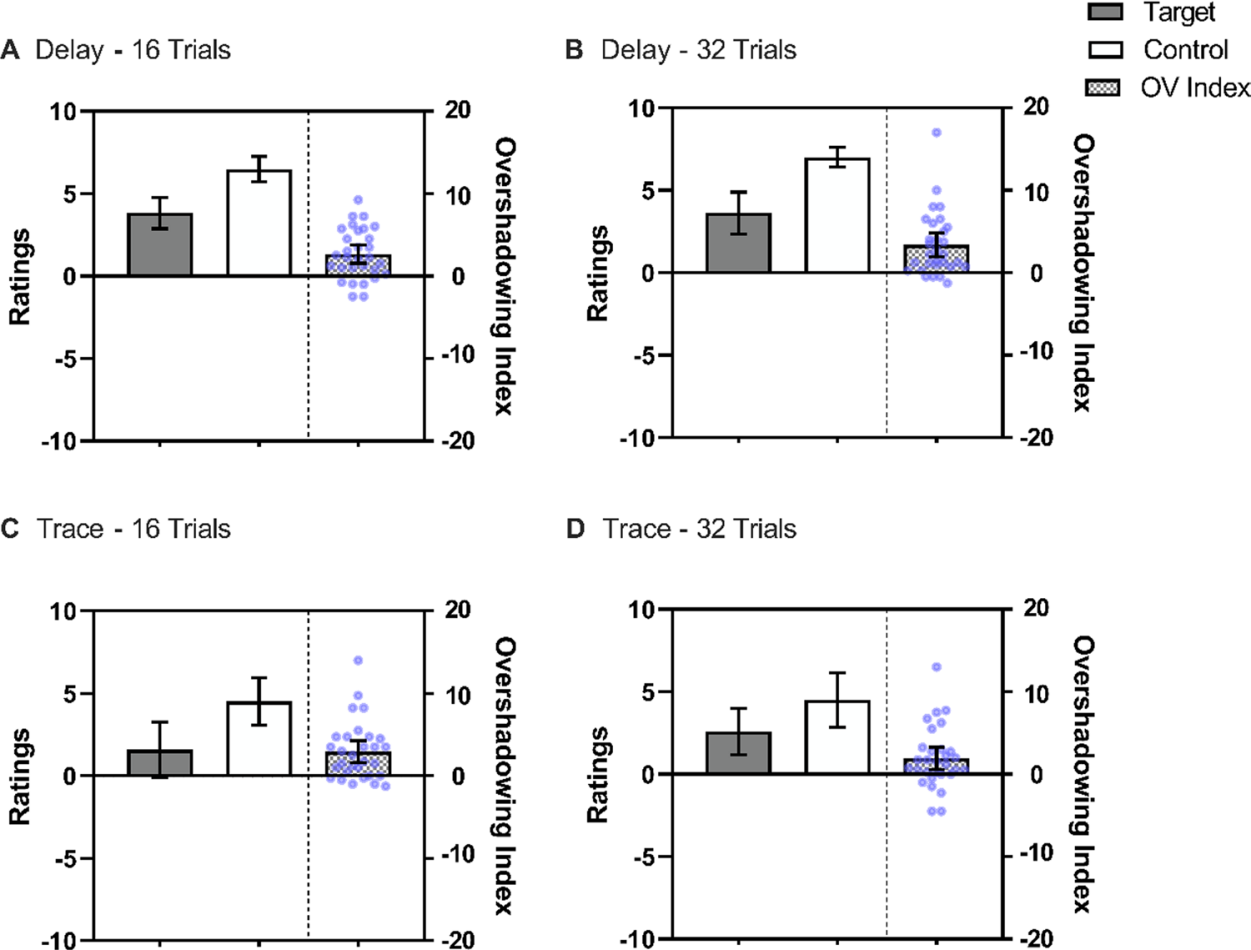
Mean ratings across streams in Experiment 2 for the Target cue which was trained as part of a compound and the Control cue which was trained alone. The checkered bars represent the Overshadowing Index (Control Cue – Target Cue); values above zero indicate Overshadowing and a value of 0 represents no competition. The blue points represent each participant’s Overshadowing Index. The upper row displays ratings in the Delay conditions; Panel **A** for 16 trials and Panel **B** for 32 trials. The lower row displays ratings in the trace conditions; Panel **C** for 16 trials and Panel **D** for 32 trials. Error bars represent 95% confidence intervals

The left part of each panel in Fig. 3 illustrates the mean ratings for each experimental condition. In general, ratings in the trace conditions were lower than in the delay conditions, suggesting an effect of contiguity. As in Experiment 1, the overshadowing indices were above 0 in all conditions, suggesting overshadowing irrespective of the number of trials and contiguity. A 2 (Trials: 16 vs. 32) × 2 (Contiguity: Delay vs. Trace) × 2 (Cue: Control vs. Target) repeated-measures ANOVA revealed a main effect of Cue, *F*(1, 29) = 26.88, *p* < .001, η^*2*^_*p*_ = .48, 95% CI [.20, .65], a main effect of Contiguity *F*(1, 29) = 9.37, *p* < .05, η^*2*^_*p*_ = .24, 95% CI [.03, .46], and a three-way interaction *F*(1, 29) = 4.86, *p* < .05, η^*2*^_*p*_ = .14, 95% CI [<.01, .37]. No other main effect or interaction was significant, *F* for the main effect of Trial, *F*(1, 29) = 2.99, *p* = .094. Subsequent analyses explored the critical Cue × Contiguity interaction in each level of Trial.

In the 16-trial conditions, there was a main effect of Cue *F*(1, 29) = 28.84, *p* < .001, η^*2*^_*p*_ = .50, 95% CI [.22, .66] and Contiguity, *F*(1, 29) = 10.36, *p* < .05, η^*2*^_*p*_ = .26, 95% CI [.04, .48], but no interaction, *F*(1, 29) = 0.21, *p* = .652.

In the 32-trial conditions, there was an effect of Cue, *F*(1, 29) = 20.09, *p* < .001, η^*2*^_*p*_ = .41, 95% CI [.13, .59], Contiguity, *F*(1, 29) = 6.75, *p* < .05, η^*2*^_*p*_ = .19, 95% CI [.01, .41], and interaction, *F*(1, 29) = 4.22, *p* = .049, η^*2*^_*p*_ = .13, 95% CI [<.01, .35]. Further analyses of this interaction showed that overshadowing was reliable in both conditions, *t*(29) = 4.77, *p* < .001, *d* = 0.87, 95% CI [0.44, 1.28], and *t*(29) = 2.84 = *p* < .05, *d* = 0.52, 95% CI [0.13, 0.90], for delay and trace conditioning, respectively. Note that the effect size for overshadowing in the case of Trace was lower compared to Delay. However, a comparison of the Overshadowing Index between delay and trace conditioning did not reveal significant differences in the magnitude of overshadowing, *t*(29) = 1.14, *p* = .267, *d* = 0.21.

An interesting result of Experiment 2 was that a clear contiguity effect was observed between the delay and the 500-ms trace conditions, replicating the main finding observed in the preliminary experiment. Although throughout the literature the most common effect of weakening contiguity is to produce a decrement in the behavioral control, there are many instances in which no clear difference between delay and trace conditioning procedures has been observed, even when using trace intervals larger than the one we used (Allan et al.,^2003^; Buehner & May, 2004; Foerde & Shohamy, 2011; Grillon et al., 2008; Lovibond et al., 2011). However, other experiments, in particular those aimed at detecting perceptual causal relationships, have found an effect of contiguity even with differences in interstimulus interval shorter than the 500 ms used here (e.g., Straube & Chatterjee, 2010; Walton et al., 2013). These discrepancies suggest that the overall effect of contiguity depends on the specific conditions of the learning protocol and testing. The fact that we observed sensitivity to contiguity in this experiment (and in the preliminary experiment) suggests that our procedure was adequate to explore interactions of the effects of compound conditioning with temporal contiguity.

Concerning cue competition, the present results replicated the overshadowing effect found in Experiment 1, again without detecting differences in the magnitude of overshadowing as a function of the number of trials. Despite no significant differences between delay and trace conditioning were detected, the nonsignificant tendency was in the expected direction, that is, reduced competition in the case of trace conditioning. It could be argued that the length of the trace was not long enough to reveal a change from competition to facilitation (Urcelay & Miller, 2009). Hence, the next experiment further explored the possibility of an interaction between contiguity and overshadowing but increased the length of the trace interval to 800 ms.

## Experiment 3

Experiment 2 revealed main effects of both contiguity and overshadowing. Although at a descriptive level overshadowing tended to be weaker in the trace conditions (especially with 32 training trials), there was still a clear overshadowing effect. One possibility is that the trace interval used in Experiment 2 was not long enough to yield the interaction, but was sufficient to produce a contiguity effect. For instance, Urcelay and Miller (2009, Experiment 1) found that a 10-s trace interval attenuated the overshadowing effect, but potentiation was only revealed by increasing the trace to 20 s. Thus, in Experiment 3 we increased the length of the trace interval to 800 ms and increased the ITI to 1,200 ms to maintain an appreciable ratio of waiting time in the presence of context alone relative to the control/target cue. Additionally, we removed the conditions with 16 trials, focusing only on the conditions with 32 trials because, as Experiment 2 revealed, this condition seemed more sensitive to the interaction of contiguity and compound training.

### Method

#### Participants

Forty participants (20 females and 20 males) were recruited from MTurk. Their mean age was 34.7 years, SD = 5.74 (min = 26, max =48). The same recruitment criteria as in previous experiments were applied. Participants received US$5.50 as compensation.

One participant acknowledged not paying attention during the task and 14 participants failed to respond correctly on the attentional check. These participants were eliminated from the experiment. Hence, 25 participants provided data for the analyses (11 females and 14 males; mean age = 34.68 years; *SD* = 5.72).

#### Design and procedure

A within-subjects design with 2 (Contiguity: Delay vs. Trace) × 2 (Cue: Control vs. Target) was used. The procedure was similar to Experiment 2, except that all streams contained 32 training trials, the length of the trace interval was increased to 800 ms, and the length of ITI was increased to 1,200 ms in all streams.

The overall number of streams in this experiment was 30. There were 12 streams in which the control cue was tested, 12 streams in which the target cue was tested, and six streams in which the overshadowing cue was tested. The 30 streams were divided into three blocks of ten streams (see OSM). Each stream lasted 56 s in the delay condition and 88 s in the trace condition.

### Results

**Fig. 4:**
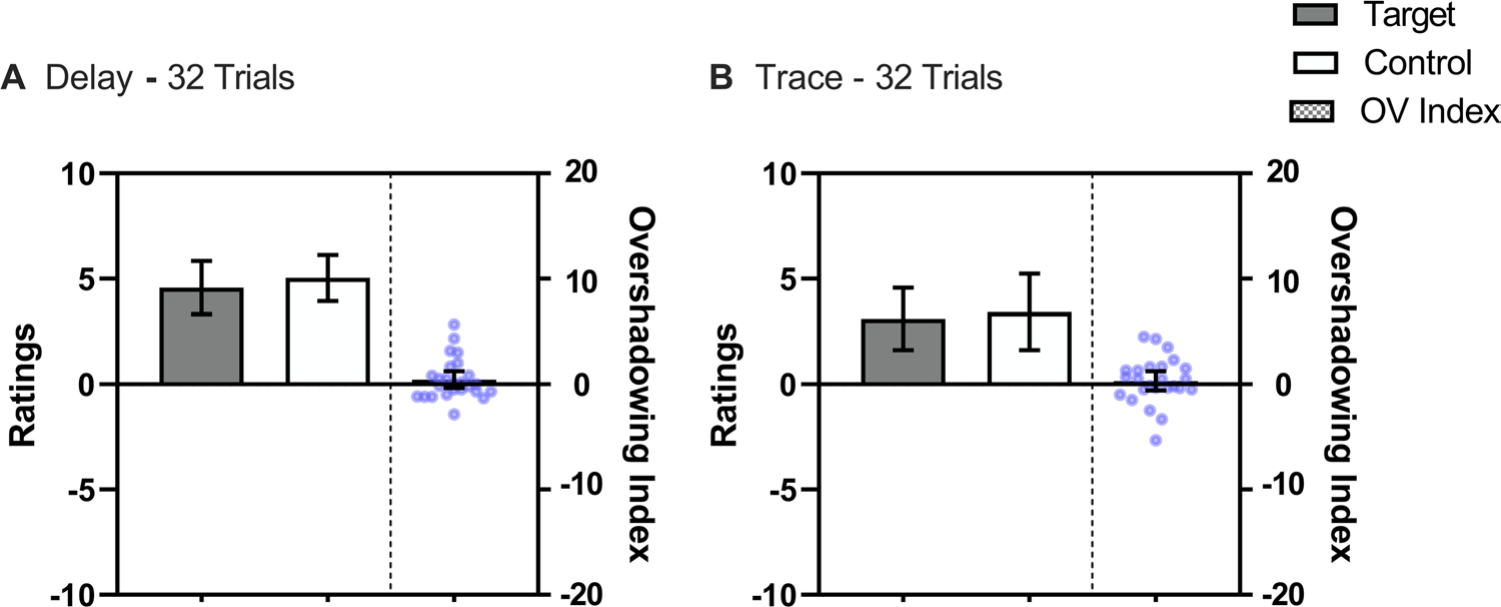
Mean ratings across streams in Experiment 3 for the Target cue, which was trained as part of a compound, and the Control cue, which was trained alone. The checkered bars represent the Overshadowing Index (Control Cue – Target Cue); values above zero indicate Overshadowing and a value of 0 represents no competition. The blue points represent each participant’s Overshadowing Index. Panel **A** displays the ratings for delay conditioning and Panel **B** for trace conditioning. Error bars represent 95% confidence intervals

The results of Experiment 3 are depicted in Fig. 4, which suggests that trace conditions received lower ratings than delay conditions. However, unlike the previous experiments, the Overshadowing Index was close to zero, in that the control and target cues received similar ratings, suggesting no overshadowing. A repeated-measures 2 (Contiguity: Delay vs. Trace) × 2 (Cue: Control vs. Target) ANOVA confirmed these impressions, revealing a main effect of Contiguity *F*(1, 24) = 5.44, *p* < .05, η^*2*^_*p*_ = .18, 95% CI [<.01, .42], and no main effect of Cue, *F*(1, 24) = 2.43, *p* = .132, nor an interaction between these factors *F*(1, 24) = 0.03, *p* = .85.

Unexpectedly, the overshadowing effect was much weaker in the delay condition relative to previous experiments. Clearly, some of the changes implemented in this experiment appreciably reduced overshadowing. Several speculative thoughts can be advanced to explain the weaker overshadowing observed in Experiment 3. We implemented two main changes from Experiment 2 in Experiment 3. First, we increased the trace interval and also increased ITI. Because in a delay condition the change in the trace length should have had no impact, it is the increase in the ITI that likely produced the attenuation of overshadowing in delay conditions.

It is well known that increasing the ITI (i.e., spaced trials) enhances behavioral control, at least for cues trained alone (e.g., Mercier & Parr, 1996). In light of this, the increase of the ITI might have provided a better scenario for participants to process each trial, and in particular, the target cue that was trained in the presence of the overshadowing cue. That is, in the prior experiments, the use of a shorter ITI may have attenuated the processing of the cues, and this “attentional blink” (Nieuwenstein et al., 2009) might have been particularly pronounced in the detection of the target cue in compound cue trials. Note that the greater physical salience of the overshadowing cue might have recruited attentional resources of participants towards the overshadowing cue, thereby reducing processing of the target cue. This may have in turn degraded the perception of relatedness between the target cue and outcome in previous experiments. However, the use of a longer ITI in Experiment 3 may have allowed for better attention to the target cue in compound cue trials and hence reduced the overshadowing effect.

Examination of the animal literature, however, reveals a pattern that seems opposite to the one just described. That is, longer ITIs tend to increase the overshadowing effect (e.g., Stout et al., 2003b). Moreover, using the streamed-trial procedure, Hannah et al. (2009) manipulated the length of ITI (100 vs. 250 ms) and did not find differences in the magnitude of blocking. Given the differences in terms of procedures and species as well as values of the ITI, it is difficult to draw a clear conclusion based on the previous literature. However, comparing Experiments 2 and 3, it seems clear that the change in the ITI produced an improvement in the detection of relatedness between the target cue and the outcome, which reduced overshadowing. For instance, Murphy et al. (2022) found that the duration of the cue influenced the degree of the cue-outcome relationship in the streamed-trial procedure. That is, longer exposure to cues increased the perception of relatedness. However, this effect was less effective compared to the frequency of trials, suggesting that the repetition of trials was the key factor determining the detection of relatedness. In our case, longer ITIs might have enhanced the degree of relatedness, particularly of the target cue, and consequently, decreased the magnitude of overshadowing.

A final consideration is that longer ITIs increase the overall length of all streams. This increase might cause the participant´s attention to wander during some of the streams. This in turn may have led participants to perceive all streams as more similar and consequently have resulted in more uniform ratings across streams and conditions, resulting in reduced sensitivity to the manipulations. Notably, in this experiment, we found a weaker effect of contiguity, even in the condition in which the trace interval was 300 ms longer than in Experiment 2. Unfortunately, we are unable to track participants’ behaviour during the presentation of the streams, but this interpretation seems plausible given the online setting without active responses being requested within relatively long streams of trials.

Based on these considerations and on the finding that the longer ITI used in Experiment 3 attenuated overshadowing, in Experiment 4 we further explored the interaction between contiguity and overshadowing but reduced the number of training trials to 12. So far, we had observed no differences in the degree of overshadowing as a function of the number of training trials; however, prior studies of cue competition suggested that a smaller number of training trials might promote overshadowing (Bellingham & Gillette, 1981; Reed & Quigley, 2019; Stout et al., 2003a). By reducing the number of trials, we avoided the potential shortcoming described above concerning the use of a long stream on the detection of relatedness in a rapid stream of trials. Additionally, we increased the length of the trace to 1,000 ms (200 ms more than in Experiment 3) to facilitate the detection of the critical interaction. Thus, we continued using a 1,200-ms ITI in the next experiment. If we again failed to observe overshadowing in the delay condition, the presence of a longer ITI would be a more likely candidate to account for the attenuation of overshadowing seen in Experiment 3.

## Experiment 4

Given the absence of an overshadowing effect and the unavoidable use of long streams given the parameters used in Experiment 3, we implemented several changes in the protocol to explore the potential interaction. First, we used shorter streams of events, by presenting only 12 training trials, to make the overall duration of the streams and the experiment shorter. Second, as we suggested in the *Introduction*, increasing the demands of the task may provide a better situation for detecting the sought-after interaction of contiguity and compound training. So, we made the task harder by equating the ratio of training and filler trials (i.e., relative to previous experiments, there was a higher density of filler trials per stream). Finally, following the same rationale provided in the *Introduction* to Experiment 3, we further extended the length of the trace interval to 1,000 ms (200 ms longer than in Experiment 3). All these changes were implemented to increase sensitivity to cue competition and facilitation.

### Method

#### Participants

Forty participants (18 females and 22 males) were recruited from mTurk. Their mean age was 35.61 years, SD = 6.10 (min = 23, max = 46). The same recruitment criteria as in the previous experiments were applied. Participants received US$3.50 as compensation. One participant acknowledged not paying attention during the task and 13 participants failed to respond correctly on the attentional check. Hence, 26 participants were considered for subsequent analyses (ten females and 16 males; mean age = 35.46 years; *SD* = 6.02).

#### Design and procedure

A within-subjects 2 (Contiguity: Delay vs. Trace) × 2 (Cue: Control vs. Target) design was used. The procedure was identical to Experiment 3 except that the number of training trials was reduced to 12, the number of filler trials was increased to 12, the trace interval was increased to 1,000 ms, and in the compound condition, the overshadowing cue was tested the same number of times as the target cue.

There were 24 streams. On eight streams the control cue was tested, on eight streams the target cue was tested, and on eight streams the overshadowing cue was tested. The 24 streams were divided into two blocks of 12 streams (see OSM). The duration of each stream was 19 s for the delay condition and 43 s for the trace condition.

### Results

**Fig. 5:**
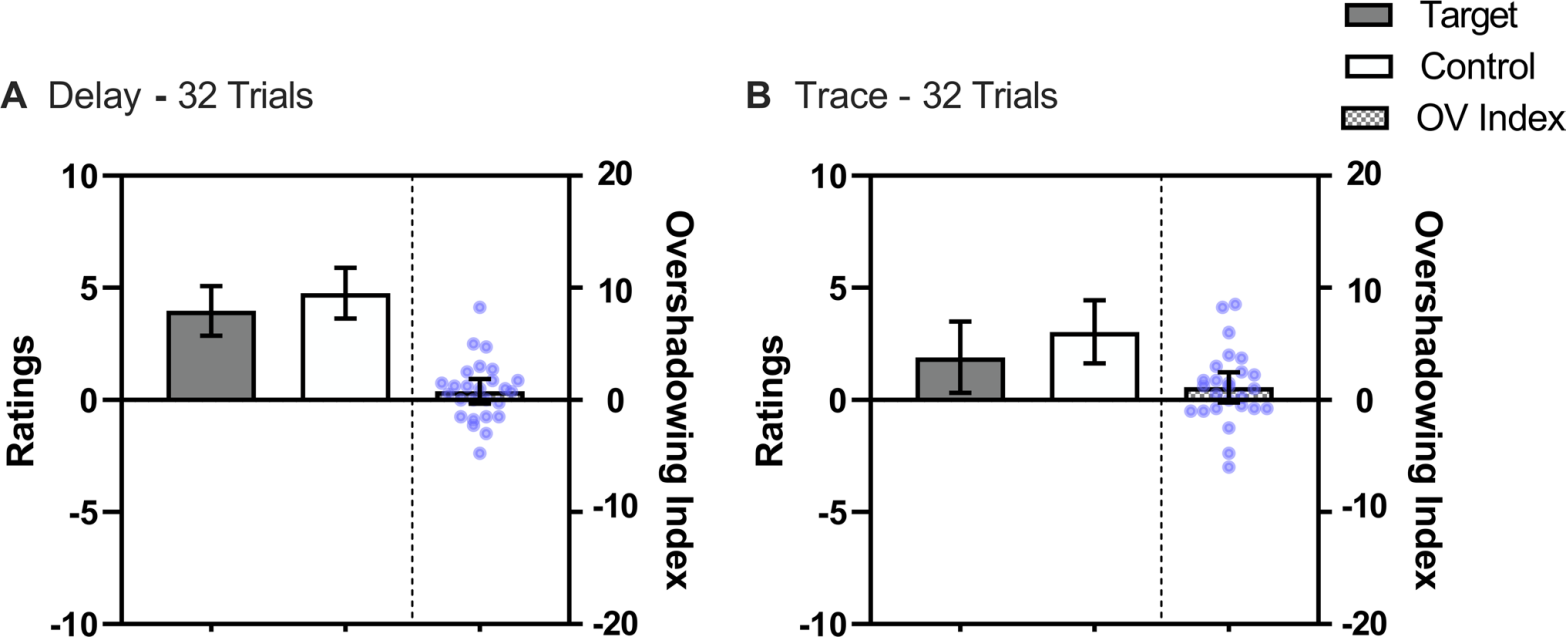
Mean ratings across streams in Experiment 4 for the Target cue, which was trained as part of a compound, and the Control cue, which was trained alone. The checkered bars represent the Overshadowing Index (Control Cue – Target Cue); values above zero indicate Overshadowing, a value of 0 reflects no competition. The blue points represent each participant’s Overshadowing Index. Panel **A** displays the ratings for delay conditioning and Panel **B** for trace conditioning. Error bars represent 95% confidence intervals

The results of Experiment 4 are depicted in Fig. 5, which suggests both contiguity and overshadowing effects, but no interaction. A repeated-measures 2 (Contiguity: Delay vs. Trace) × 2 (Cue: Control vs. Target) ANOVA revealed an effect of Contiguity *F*(1, 25) = 8.10, *p* < .05, *η*^*2*^_*p*_ = .24, 95% CI [.02, .47], and Cue *F*(1, 25) = 4.26, *p* < .05, *η*^*2*^_*p*_ = .15, 95% CI [<.01, .38], but no Contiguity × Cue interaction, *F*(1, 25) = 0.22, *p* = .642.

Experiment 4 replicated to a large extent the findings of Experiment 3, in which an effect of contiguity and a much weaker effect of overshadowing were observed. Similar to Experiment 3, the ITI was 1,200 ms, twice the length of that used in Experiments 1 and 2, in which we found a stronger overshadowing effect. Like Experiment 3, the present experiment suggests that the increase in ITI in the rapid trial procedure may have nonlinear effects, and a longer time between trials may have reduced overshadowing (c.f., Stout et al., 2003a). Although the implications that different ITIs affect the magnitude of overshadowing are interesting, they are beyond the scope of the present series, which aimed to explore the interaction between overshadowing and contiguity. Hence, in Experiment 5 we restored the ITI duration that was used in Experiments 1 and 2.

## Experiment 5

A reliable overshadowing effect was observed in Experiments 1 and 2. In those experiments, trials were separated by a short ITI (600 ms). In contrast, in Experiments 3 and 4, overshadowing was attenuated when a longer ITI (1,200 ms) was used. Hence, in Experiment 5 we reverted to the parameters used in Experiment 2 (trace interval = 500 ms and ITI = 600 ms), which proved sensitive to detect the main effects of both contiguity and overshadowing, but with some additional changes aimed at increasing the demands of the task.

The same number of training trials (12) and filler trials (12) were administered as in Experiment 4. However, in Experiment 5, we also included streams with a low cue-outcome contingency (Δp = 0.25). This was done for three reasons. First, as we were using a small number of target trials (12), possibly participants were not sufficiently sensitive to the cue-outcome relationship. Note that 12 training trials is a lower value than prior research using the streamed-trial procedure (e.g., Crump et al., 2007; Hannah et al., 2009). The presence of different streams with high and low numbers of trials also allowed us to implement an awareness-sensitivity check to ensure that participants were able to discriminate between different levels of contingencies. Consequently, differences in the magnitude of overshadowing, if any, should have been primarily caused by the interposition of the trace interval, rather than by an impairment in the detection of contingency during the streams (see *Data exclusion*). Second, the introduction of low contingency streams increased the variability across streams, likely making streams more differentiable, thereby presumably creating a more engaging task. Third, we expected to find a reduced overshadowing effect in the streams with lower contingencies as suggested by previous studies of blocking using the streamed-trial procedure (e.g., Hannah et al., 2009; Mutter & Arnold, 2020) and a broader set of procedures and species (see Urcelay, 2017). Overall, we expected that the presence of a trace interval would at least attenuate the magnitude of overshadowing if not yield potentiation.

### Method

#### Participants

Fifty participants (17 females and 33 males) were recruited from mTurk. Their mean age was 37.44 years, SD =6.25 (min = 25, max =49). The same recruitment criteria as in the previous experiments were applied. Participants received US$3.00 as compensation.

#### Data exclusion

One participant acknowledged not paying attention during the task and was removed from the analyses. Unlike previous experiments, we applied a contingency-sensitivity check to guarantee that we only included participants who were able to discriminate between high and low contingency streams during the experiment. We compared the conditions of high and low contingency in the delay conditions that were tested on the control cue. A priori, this is the most “neutral” condition in the experiment or at least the condition in which a clear effect of contingency would most be expected. Hence, we only included participants who showed higher ratings for the control cue in the high contingency conditions compared to the control cue in the low contingency conditions (participants with identical ratings in both contingency levels were also excluded). Twenty participants did not exhibit sensitivity to this difference in contingencies. Consequently, 29 participants were included in the analyses (11 females and 18 males; mean age = 37.13 years; *SD* = 6.11).

#### Design and procedure

A within-subjects design with 2 (Contingency: High vs. Low) × 2 (Contiguity: Delay vs. Trace) × 2 (Cue: Control vs. Target) independent variables was used. The procedure was similar to Experiment 2 (i.e., a trace interval of 500 ms and an ITI of 600 ms), except that: all streams contained 12 training trials and 12 filler trials; half of the streams were programmed with high cue-outcome contingency during training (Δp = 0.75; nine type ‘a’ trials and three type ‘b’ trials), and the other half of the streams with low cue-outcome contingency (Δp = 0.25; three type ‘a’ trials and nine type ‘b’ trials). The contingency was Δp = 0.67 for the single- and compound-filler trials (four type ‘a’ trials and two type ‘b’ trials).

There was a total of 40 streams in the experiment. There were 16 streams in which the control cue was tested, 16 streams in which the target cue was tested, and eight streams in which the overshadowing cue was tested. The 40 streams were divided into four balanced blocks (see OSM). The duration of a delay condition stream was 19 s, and of a trace condition stream was 31 s.

### Results

**Fig. 6:**
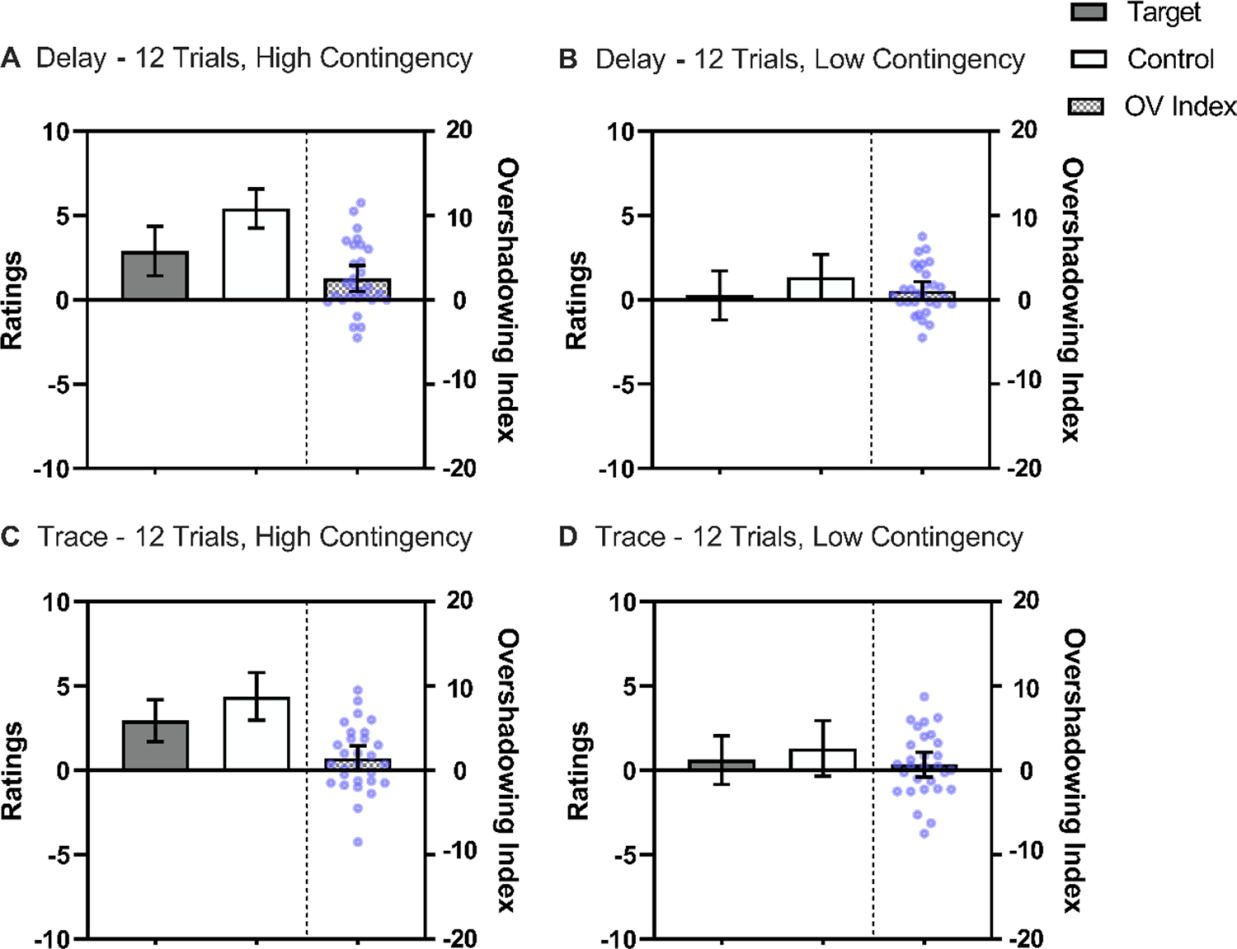
Mean ratings across streams in Experiment 5 for the Target cue, which was trained as part of a compound, and the Control cue, which was trained alone. The checkered bars represent the Overshadowing Index (Control Cue – Target Cue); values above zero indicate Overshadowing and a value of 0 represents no competition. The blue points represent each participant’s Overshadowing Index. The upper row displays ratings in delay conditioning; Panel **A** for High Contingency and Panel **B** for Low Contingency conditions. The lower row displays ratings in trace conditioning; Panel **C** for High Contingency and Panel **D** for Low Contingency conditions. Error bars represent 95% confidence intervals

Figure 6 displays the mean ratings across conditions. As expected, conditions with low contingency received lower ratings. Indicative of overshadowing, the target cue received lower ratings than the control cue. There was a weak tendency toward reduced overshadowing in the trace conditions.

A repeated-measures 2 (Contingency: High vs. Low) × 2 (Contiguity: Delay vs. Trace) × 2 (Cue: Control vs. Target) ANOVA revealed main effects of Cue, *F*(1, 28) = 8.01, *p* < .05, η^*2*^_*p*_ = .22, 95% CI [.02, .44], Contingency, *F*(1, 28) = 49.49, *p* < .001, η^*2*^_*p*_ = .64, 95% CI [.38, .76], and the Contingency × Cue interaction, *F*(1, 28) = 4.99, *p* < .05, η^*2*^_*p*_ = .15, 95% CI [<.01, .38]. However, neither the main effect of Contiguity *F*(1, 28) = 0.18, *p* = .673, the Contiguity × Cue interaction, *F*(1, 28) = 1.46, *p* = .239, nor the three-way interaction, *F*(1, 28) = 0.55, *p* = .463, were significant.

Pooling over delay and trace conditions, analyses of the Contingency × Cue interaction found that overshadowing was significant in the High contingency conditions, *F*(1, 28) = 10.95, *p* < .05, η^*2*^_*p*_ = .28, 95% CI [.04, .50], but not in the Low contingency conditions, *F*(1, 28) = 2.60, *p* = .102. Therefore, overshadowing was attenuated by degrading the cue-outcome contingency, which is in line with the view that degrading contingency reverts competition into facilitation (see Urcelay, 2017), or at least attenuates competition (e.g., Hannah et al., 2009; Mutter & Arnold, 2020).

Overall, in Experiment 5, a reliable effect of overshadowing was observed. Moreover, weakening cue-outcome contiguity seemed to attenuate overshadowing, although we did not find statistically significant evidence of an interaction between contiguity and overshadowing nor a clear effect of contiguity. The absence of an overall contiguity effect may have been driven by the particular influence of the trace interval in the compound streams. Looking at Fig. 6, one can observe similar ratings of the target cue in the trace condition compared to the delay condition with high contingency, and a [weak] tendency toward the ratings being higher ratings with low contingency. However, this tendency was not large enough to produce a statistically significant interaction.

## General discussion

In five experiments, we explored contiguity and overshadowing effects as well as their potential interaction using a rapid stream of trials procedure. Both overshadowing and contiguity effects were observed across most experiments. On one hand, the target cue trained in compound with a more salient cue received lower ratings than the control cue trained alone (Experiments 1, 2, 4, and 5), demonstrating the overshadowing effect. On the other hand, inserting a temporal gap of 500 ms (Experiments 2 and 5), 800 ms (Experiment 3), or 1,000 ms (Experiment 4) weakened the rated strength of relatedness between cues and outcomes, revealing the expected effect of contiguity. However, trace conditioning did not appreciably modulate the magnitude of overshadowing across experiments.

Contrary to our expectations, an interaction between the two factors (overshadowing and trace conditioning) was not consistently observed. The current protocol and experimental parameters may not have been ideal to observe a shift from competition to no-interaction (e.g., Herrera et al., 2022; Alcalá et al., under review), or to facilitation (Alcalá et al., 2023; Batsell et al., 2012; Cunha et al., 2015; Schachtman et al., 1987; Urcelay & Miller, 2009). Several tentative explanations can be speculated upon to account for the lack of a reliable effect of trace on overshadowing, but before that, we would like to highlight two relevant findings of this experimental series.

One salient finding in this series is that, across experiments, a reliable overshadowing effect was observed. This is the first demonstration of overshadowing in the context of the rapid stream procedure, extending the blocking effect previously reported (e.g., Hannah et al., 2009). A priori, we expected to find a reduction of overshadowing with an increase in the number of trials within a stream; however, we observed overshadowing irrespective of the number of training trials (16 vs. 32 in Experiments 1 and 2). Research with nonhuman animals examining overshadowing has revealed that an increase in the number of trials often attenuates overshadowing (Bellingham & Gillette, 1981; Stout et al., 2003a; also see Reed & Quigley, 2019, for a similar observation in humans). In contrast, the present absence of sensitivity of overshadowing to the number of training trials is consistent with other results in humans (see Herrera et al., 2022), which found overshadowing to be independent of the number of training trials (four, eight, or 16). Unlike the number of trials, overshadowing in Experiments 3 and 4 was attenuated when the ITI used was 1,200 ms (twice the duration of the ITI in the other experiments). This pattern of results is inconsistent with data from rodents suggesting that spaced trials (i.e., longer ITIs) tend to increase the magnitude of overshadowing (Stout et al., 2003b). We provided a few tentative explanations for these somewhat contradictory results in the *Discussion* of Experiments 3 and 4, although the differences between protocols and species make it difficult to reconcile the discrepant results. Moreover, in Experiments 3 and 4, we observed reduced overshadowing regardless of cue-outcome temporal contiguity. It could be that longer intervals between trials facilitated learning about the target cue (trained in compound). In the streamed-trial procedure, longer cue exposure on each trial increases the perception of relatedness between events (e.g., Murphy, et al., 2022). Nevertheless, this interpretation is based on cross-experiment comparisons in which other factors (such as the number of trials) were also manipulated. Therefore, future studies should properly assess whether long ITIs attenuate overshadowing in this procedure. Whatever the merits of this interpretation, these experiments potentially serve as a starting point for future studies exploring the effect of different ITIs when cues are trained as part of a compound, given that most of the published studies manipulating this parameter used nonhuman subjects (e.g., Stout et al., 2003b).

A second relevant outcome is that a contiguity effect was observed in our adaption of the streamed-trial procedure. To our knowledge, this is the first time that contiguity has been systematically assessed using the streamed-trial procedure. Note that several learning protocols in humans have shown little sensitivity to contiguity manipulations, even when using longer trace intervals (e.g., Allan et al., 2003; Buehner & May, 2004; Foerde & Shohamy, 2011; Grillon et al., 2008; Lieberman et al., 2008; Lovibond et al., 2011). The fact that in our preparation a contiguity effect was obtained using relatively short trace intervals (ranging from 500 to 1,000 ms) opens an avenue for investigating trace conditioning phenomena more efficiently than prior paradigms, such as trial-by-trial predictive learning tasks (i.e., the allergy task). That contiguity manipulations can be implemented in an adaptation of the streamed-trial procedure is in line with one of the goals of the seminal study by Crump et al. (2007), which was to reduce the length of experiments in a highly controlled preparation that enables the manipulation of different variables using within-subject experimental designs.

As previously noted, we did not observe the expected crossover interaction between contiguity and overshadowing effects. This pattern is inconsistent with results with animals (e.g., Urcelay & Miller, 2009) and also some recent results with human participants (Alcal﻿á et al., 2023; Cunha et al., 2015). Nevertheless, the fact that in Experiments 2 and 5 the reported effect size for overshadowing was numerically lower in trace conditioning compared to delay conditioning is somewhat consistent with the idea that competition weakens (and perhaps transitions into facilitation) as a direct function of contiguity. However, this effect in the streamed-trial procedure is less robust than in other types of human learning paradigms exploring this interaction (Alcalá et al., 2023; Alcalá et al., under review; Cunha et al., 2015; Herrera et al., 2022). We suggest that there are at least four potential reasons for the absence of a cross-over interaction in the present series.

First, the use of different geometrical shapes and the pseudo-random alternation between left and right positions of the cues might have promoted elemental processing of the cues. Some accounts of facilitation rely on the engagement of configural processing (Herrera et al., 2022; Urcelay & Miller, 2009), whereas our protocol, which perceptually presents the two cues side by side but separated by a space, may have promoted elemental processing of the cues and thus favored cue competition (Williams et al., 1994), resulting in an overshadowing effect across the contiguity manipulations. Flexible (i.e., elemental and configural) encoding has been proposed as one critical determinant of competition or facilitation (Urcelay & Miller, 2009), and importantly the type of encoding strategy used presumably depends on several different variables and parameters (see Melchers et al., 2008). For instance, pre-training manipulations that encourage configural processing appear to eliminate overshadowing in animals (Urcelay & Miller, 2009), and also reduce blocking in humans (e.g., Williams et al., 1994, Experiment 4). Similarly, pre-training on a discrimination that requires a configural solution facilitates patterning discrimination or biconditional discrimination, in which configural processing is critical to learn the new discrimination (Mehta & Russell, 2009). Conversely, pre-training (with different cues) that promotes elemental encoding of the stimuli facilitates blocking (William et al., 1994). Another variable that may determine how stimuli are processed is the spatial distance between them in the layout, resulting in differences in cue-competition phenomena. For example, when predictors were placed close to each other (promoting configural processing), blocking was attenuated (e.g., Glautier, 2002; Livesey & Boakes, 2004). In our experiments, the continuous alternation between left-right positions during the streams might have prevented the encoding of the stimuli in a configural manner, promoting instead elemental processing. Overall, in the current experiments, the nature of the stimuli and task may have promoted the use of an elemental encoding strategy, which in turn may have impaired observation of facilitation.

Second, the duration of the trace interval seems to be a critical factor in determining the outcome of cue interactions. For instance, in Urcelay and Miller (2009) a 20-s trace interval (but not a 10-s trace interval) resulted in potentiation of conditioned responding to the target cue (also see with a different timescale, Batsell et al., 2012). Translated to our experiments, the trace intervals used in the present series might be not long enough to yield potentiation. Recently, Herrera et al. (2022) proposed that the decay of stimulus representation (and the concomitant loss of potential to enter into associative strength) as the trace interval increases may interact with the stimulus generalization in a modification of Pearce’s (1987) configural theory. According to this model, a decrease in the associative strength is accompanied by an increase in generalization from training to test (formally operationalized as an increase in the shared elements between the two stimuli that make the compound [target cue + overshadowing cue] and the test stimulus [target cue]), leading to less generalization decrement and hence reduced competition. However, the very short trace used in the current experiments might not have been long enough to produce a significant decrement in the associative strength of the cues, mitigating this expected asymmetrical effect.

Following a reviewer’s recommendation, we consider a third possibility. Instructions for the task were framed in terms of both contingency – “the shape predicted the impending appearance of the picture,” and contiguity – “the shape was soon followed by presentation of the picture.” In Experiment 2, we reported a Cue × Contiguity interaction; however, overshadowing was reliable in both temporal conditions (albeit reduced in trace conditioning). Given a sufficient number of trials (i.e., 32) with a fixed contingency (i.e., Δp = 0.75), participants might be biased toward using the information stemming from the cue-outcome temporal relationship. In other words, in the absence of contingency manipulations, the manipulation of contiguity may have been more salient. However, in Experiment 5, we used a smaller number of trials (only 12) and added two different levels of contingency. Participants may have based their judgments predominantly on the contingency relationship, leaving little room to deploy resources for processing the temporal interval. Assuming this interpretation, it is not surprising that in Experiment 5 the effect of contiguity was not reliable, neither alone nor in interaction with other factors. Indeed, it is the only experiment of the experimental series in which the contiguity effect was not significant. Moreover, we observed asymmetric overshadowing as a function of the Contingency: overshadowing was attenuated in the Low-Contingency condition. This result is congruent with the view that degrading the cue-outcome contingency can also attenuate competition (see Urcelay, 2017). To some extent, this suggests that participants might have better processed the contingency relationship relative to the temporal relationship in this last study. Framing the task primarily in terms of contiguity may have revealed a different pattern of results.

Fourth, decay of information (a.k.a., *trace decay theory*) is one of the more common accounts for behavioral deficits observed in trace conditioning paradigms. When a stimulus no longer stimulates a given sensory system, neural activity (putatively a representation of the stimulus) putatively decays as a function of time passed, decreasing the stimulus’ capacity to enter into an association with the outcome (e.g., Rawlins, 1985). As an alternative to trace decay theory, a large volume of research and several theories assume that intervening events occurring during the trace interval become associated with the outcome, and these associations interfere with the expression of the target cue-outcome association, producing a decrement in the behavioral response to the target cue (Costa & Boakes, 2011; Lagnado & Speekenbrink, 2010; Lieberman, Carina, Vogel, & Nisbet, 2008; Revusky, 1971). In this experimental series, we used a “*clean*” trace, without any nominal intervening events during the trace interval as was done previously in animal research (Batsell et al., 2012; Urcelay & Miller, 2009). This raises the question of what effect if any would overt intervening events have on the relatedness judgments if these events occurred during the trace interval, and whether this configuration might promote competition or facilitation.

Overall, the current results help to establish boundary conditions in the search for general principles of learning. Basic learning phenomena such as overshadowing and blocking are thought to be domain-general and widely observed across different learning domains and species. Unfortunately, this assumption long went unexamined (e.g., see Telga, Alcalá, Heyes, & Urcelay, 2023, for the first demonstration of overshadowing in social learning with humans), and now growing evidence suggests that it is an oversimplification (e.g., Maes et al., 2016). We think that it is important to establish the boundary conditions for cue competition phenomena, that is, to determine which preparations and parameters hinder or facilitate the observation of competition. Indeed, some behavioral effects that are opposite to competition, such as potentiation and augmentation, suggest that these may depend on a narrower set of parameters than overshadowing or blocking. But the fact that facilitation phenomena seem to occur under at least a select set of parameters should not undermine their importance. Ultimately, a key goal is to determine their boundary conditions in order to develop theories that account for these discrepant findings. Since the first evidence of blocking in humans (see Dickinson et al., 1984), numerous experiments have been conducted to study the determinants of cue interactions in humans. As we briefly described in the *Introduction*, there is a long list of behavioral variables that modulate cue interactions. However, we note that most of the relevant studies focused on blocking, which seems to involve associative interference as well as cue competition (Miguez & Miller, 2022). Consequently, studies investigating the overshadowing effect, such as the present one, are scarcer and might help to shed light on the nature of cue competition and its generality.

In summary, the results of the current study provided good evidence of contiguity and overshadowing effects in the context of the streamed-trial procedure. However, the effect of temporal contiguity in modulating overshadowing was not reliable, although in some experiments a nonsignificant tendency of overshadowing to be inversely related to contiguity was seen. Therefore, other variables beyond contiguity also modulate cue competition. Determining the specific variables and their interplay is critical to further advance knowledge concerning the specific mechanisms of learning that are engaged when there are multiple cues.

### Supplementary Information

ESM 1 (DOCX 27 kb)
