# Supplementary material for: Contiguity and overshadowing interactions in the rapid-streaming procedure
Source: Learn Behav. 2023 Apr 17;51(4):482–501. doi: 10.3758/s13420-023-00582-4 (PMC10716097; doi:10.3758/s13420-023-00582-4)
Supplement: Supplementary file 1 — (DOCX 27 kb) [file 13420_2023_582_MOESM1_ESM.docx]

**Contiguity and Overshadowing Interactions in the Rapid-Streaming Procedure**

José A. Alcalá^12^, Ralph R. Miller^3^, Richard D. Kirkden ^4^, Gonzalo P. Urcelay^1^

*Supplementary Material*

Breakdown of the number of streams in each experimental condition and the Mean and SD for the experimental conditions analysed in each experiment.

**Experiment 1**

*Table1a*

| Streams Breakdown | 16-Trials Delay | 32-Trials Delay |
| --- | --- | --- |
| Control Cue | 10 (2) | 10 (2) |
| Target Cue | 10 (2) | 10 (2) |
| Overshadowing Cue | 5 (1) | 5 (1) |

*There were five block of training and each block contained 10 streams. In brackets the number of each type of stream per block.*

*Table1b*

| Trials | | Cue | | Mean | | SD | |  |  |
| --- | --- | --- | --- | --- | --- | --- | --- | --- | --- |
| 16 |  | Control |  |  | 4.338 |  | 2.177 |  |  |
|  |  | Target |  |  | 2.179 |  | 2.479 |  |  |
| 32 |  | Control |  |  | 5.566 |  | 2.286 |  |  |
|  |  | Target |  |  | 3.328 |  | 3.186 |  |  |

**Experiment 2**

*Table 2a*

| Streams Breakdown | 16-Trials Delay | 32-Trials Delay | 16-Trials Trace | 32-Trials Trace |
| --- | --- | --- | --- | --- |
| Control Cue | 4 (1) | 4 (1) | 4 (1) | 4 (1) |
| Target Cue | 4 (1) | 4 (1) | 4 (1) | 4 (1) |
| Overshadowing Cue | 2 (*) | 2 (*) | 2 (*) | 2 (*) |

*There were four block of training and each block contained 10 streams. In brackets the number of each type of stream per block. * means than in each block appear one stream with delay and other stream with trace testing the overshadowing cue.*

*Table 2b*

| Timing | | Cue | | Trials | | Mean | | SD | |  |
| --- | --- | --- | --- | --- | --- | --- | --- | --- | --- | --- |
| Delay |  | Control |  | 16 |  | 6.493 |  | 2.037 |  |  |
|  |  |  |  | 32 |  | 7.008 |  | 1.611 |  |  |
|  |  | Target |  | 16 |  | 3.825 |  | 2.564 |  |  |
|  |  |  |  | 32 |  | 3.622 |  | 3.419 |  |  |
| Trace |  | Control |  | 16 |  | 4.525 |  | 3.824 |  |  |
|  |  |  |  | 32 |  | 4.500 |  | 4.450 |  |  |
|  |  | Target |  | 16 |  | 1.583 |  | 4.533 |  |  |
|  |  |  |  | 32 |  | 2.583 |  | 3.762 |  |  |

**Experiment 3**

*Table 3a*

| Streams Breakdown | 32-Trials Delay | 32-Trials Trace |
| --- | --- | --- |
| Control Cue | 6 (2) | 6 (2) |
| Target Cue | 6 (2) | 6 (2) |
| Overshadowing Cue | 3 (1) | 3 (1) |

*There were three block of training and each block contained 10 streams. In brackets the number of each type of stream per block.*

*Table 3b*

| Timing | | Cue | | Mean | | SD | |  |
| --- | --- | --- | --- | --- | --- | --- | --- | --- |
| Delay |  | Control |  |  | 5.034 |  | 2.649 |  |
|  |  | Target |  |  | 4.580 |  | 3.045 |  |
| Trace |  | Control |  |  | 3.433 |  | 4.378 |  |
|  |  | Target |  |  | 3.100 |  | 3.588 |  |

**Experiment 4**

*Table 4a*

| Streams Breakdown | 32-Trials Delay | 32-Trials Trace |
| --- | --- | --- |
| Control Cue | 4 (2) | 4 (2) |
| Target Cue | 4 (2) | 4 (2) |
| Overshadowing Cue | 4 (2) | 4 (2) |

*There were three block of training and each block contained 12 streams. In brackets the number of each type of stream per block.*

*Table 4b*

| Timing | | Cue | | Mean | | SD | |  |  |  |
| --- | --- | --- | --- | --- | --- | --- | --- | --- | --- | --- |
| Delay |  | Control |  |  | 4.760 |  | 2.811 |  |  |  |
|  |  | Target |  |  | 3.971 |  | 2.722 |  |  |  |
| Trace |  | Control |  |  | 3.038 |  | 3.461 |  |  |  |
|  |  | Target |  |  | 1.904 |  | 3.912 |  |  |  |

**Experiment 5**

*Table 5a*

| Stream Breakdown | 12-Trials- High-Delay | 12-Trials Low-Delay | 12-Trials- High-Trace | 12-Trials-Low-Trace |
| --- | --- | --- | --- | --- |
| Control Cue | 4 (1) | 4 (1) | 4 (1) | 4 (1) |
| Target Cue | 4 (1) | 4 (1) | 4 (1) | 4 (1) |
| Overshadowing Cue | 2 (*) | 2 (*) | 2 (*) | 2 (*) |

*There were four blocks of training and each block contained 10 streams. In brackets the number of each type of stream per block.*

*Table 5b*

| Contingency | | Timing | | Cue | | Mean | | SD | |  |  |
| --- | --- | --- | --- | --- | --- | --- | --- | --- | --- | --- | --- |
| High |  | Delay |  | Control |  |  | 5.422 |  | 3.040 |  |  |
|  |  |  |  | Target |  |  | 2.888 |  | 3.850 |  |  |
|  |  | Trace |  | Control |  |  | 4.379 |  | 3.699 |  |  |
|  |  |  |  | Target |  |  | 2.957 |  | 3.266 |  |  |
| Low |  | Delay |  | Control |  |  | 1.345 |  | 3.528 |  |  |
|  |  |  |  | Target |  |  | 0.267 |  | 3.839 |  |  |
|  |  | Trace |  | Control |  |  | 1.310 |  | 4.351 |  |  |
|  |  |  |  | Target |  |  | 0.617 |  | 3.800 |  |  |
